# A Microbial Cocaine Bioreporter

**DOI:** 10.3390/s24206549

**Published:** 2024-10-11

**Authors:** Anne-Kathrin Grimm, Dor Rozanes, Etai Shpigel, Liat Moscovici, Shimshon Belkin

**Affiliations:** 1 Institute of Analytical Chemistry, Chemo- and Biosensors, University of Regensburg, 93053 Regensburg, Germany; anne-kathrin.mildner@chemie.uni-regensburg.de; 2 Institute of Life Sciences, The Hebrew University of Jerusalem, Jerusalem 9190401, Israel; serinade@gmail.com (D.R.); etaish1@hotmail.com (E.S.); liatmosc@savion.huji.ac.il (L.M.)

**Keywords:** microbial bioreporters, bioluminescence, cocaine, cocaine esterase, *ben* operon, *Escherichia coli*, *Pseudomonas putida*

## Abstract

The continuous emergence of new illegal compounds, particularly psychoactive chemicals, poses significant challenges for current drug detection methods. Developing new protocols and kits for each new drug requires substantial time, effort, and dedicated manpower. Whole-cell bacterial bioreporters have been proven capable of detecting diverse hazardous compounds in both laboratory and field settings, identifying not only single compounds but also chemical families. We present the development of a microbial bioreporter for the detection of cocaine, the nervous system stimulant that is the second-most widely used illegal drug in the US. *Escherichia coli* was transformed with a plasmid containing a bacterial *luxCDABEG* bioluminescence gene cassette, activated by a cocaine-responsive signaling cascade. The engineered bioreporter is demonstrated to be a sensitive and specific first-generation detection system for cocaine, with detection thresholds of 17 ± 8 μg/L and 130 ± 50 μg/L in a buffer solution and in urine, respectively. Further improvement of the sensor’s performance was achieved by altering the nucleotide sequence of the *PBen* gene promoter, the construct’s sensing element, using accelerated site-directed evolution. The applicability of ready-to-use paper strips with immobilized bioreporter cells was demonstrated for cocaine detection in aqueous solutions.

## 1. Introduction

The constant emergence of new illegal psychoactive compounds constitutes a severe hurdle for current drug detection methods. Contemporary methodologies are mostly based on either immunoassays or chromatographic methods, such as LC-MS, and hence target only specific compounds that have been previously identified and characterized. There is thus an acute need for simple and rapid broad-spectrum test kits that will sensitively detect not only the original target but also its potential derivatives and analogs. This need may be at least partially met by the application of synthetic biology approaches to the design of novel drug-targeted whole-cell bioreporters. Such sensors can potentially report not on the chemical nature of the drug molecule, but rather on the biological effects it may exert on living cells; no molecule-based biosensor can provide this level of information nor can it be obtained by chemical analysis [1].

Whole-cell biosensors (bioreporters) are genetically engineered to emit a quantifiable signal in the presence of specific target chemicals, general stress conditions, or deleterious biological effects. They are mostly based on bacterial or yeast cells, the genetic engineering of which is often based on a molecular fusion of two DNA segments: (a) a sensing element, usually a gene promotor that is induced by the target compound(s), and (b) a reporting element, a gene or gene cassette encoding proteins with easily quantifiable activities. Considering the fact that newly developed drug derivatives often activate the same receptors as the parent compound and induce similar biological effects, whole-cell microbial biosensors have the potential to widen the drug detection toolbox. Bacterial sensors have been shown to detect a variety of compounds with accuracy and precision comparable with, and sometimes even surpassing, chemical analysis [2]. They have numerous potential applications, including monitoring of water toxicity [3] and genotoxicity [4], the detection of buried explosives [5], or the quantification of endocrine-disrupting compounds [6]. Here we report on the development of an *Escherichia coli*-based whole-cell bioreporter for cocaine: a nervous system stimulant and the second-most widely used illegal drug in the US [7,8], the effect of which on the brain, as a dopamine reuptake inhibitor, often leads to dependence and addiction.

The molecular circuit described in this communication is based on the cocaine esterase (CE) gene (*cocE*), originally identified by Brit et al. [9] in a *Pseudomonas maltophilia* strain isolated from coca leaves. CE degrades cocaine into ecgonine methyl ester and benzoic acid [9], a reaction similar to that taking place in the human liver [10] and the human blood postmortem [11]. In 2009, Gao et al. developed a variant of the bacterial protein that is stable at human body temperature [12]. This protein shows promise as a long-term protection against cocaine toxicity, potentially reinforcing the liver’s ability to deal with acute cocaine overdoses [13]. CocE has been the subject of multiple studies aimed at its use as a therapeutic intervention for cocaine abuse through intravenous treatment [14,15], in the course of which it has been demonstrated that its activity has not been hindered by body fluids such as blood.

**Figure 1:**
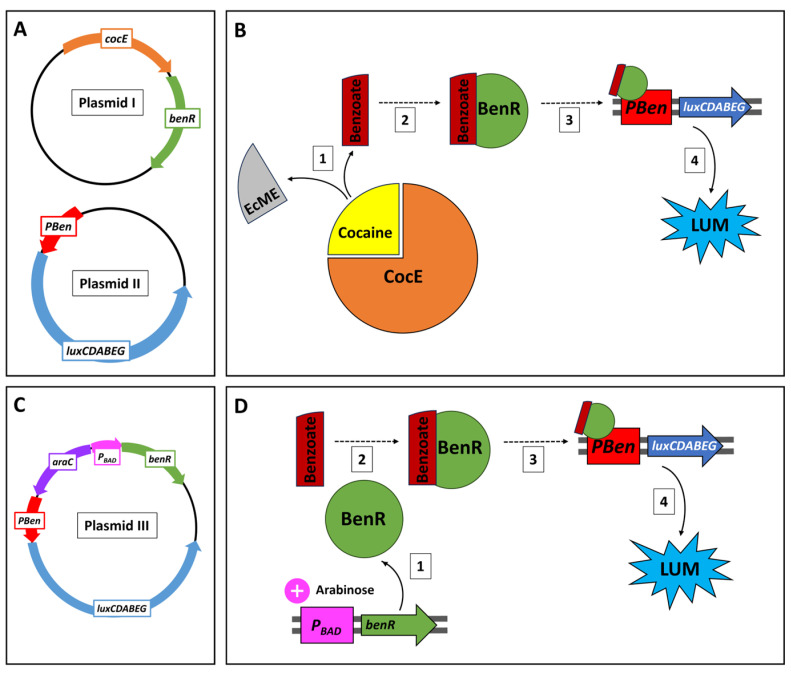
(**A**) Schemes of the two plasmids constituting the cocaine detection circuit. Plasmid I harbors the cocaine esterase gene (*cocE*) and the transcription factor *benR*. Plasmid II harbors the *P. leiognathi luxCDABEG* gene cassette controlled by the BenR-inducible promotor *PBen*. (**B**) Cocaine detection signaling cascade. Following cocaine cleavage by cocaine esterase (CocE) to ecgonine methyl ester (EcME) and benzoate (1), the latter complexes with the transcription factor BenR (2) and activates the *PBen* promotor (3). This drives the expression of the *luxCDABEG* genes, leading to a dose-dependent luminescence signal (4). (**C**) Schematic illustration of the benzoate sensor plasmid (plasmid III), which harbors the transcription factor gene *benR* along with the *P_BAD_* promoter and its regulator gene *araC*. The *luxCDABEG* gene cassette is under the control of *PBen*. (**D**) In the presence of arabinose, *benR* is expressed (1); the BenR protein forms a complex with a benzoate molecule (2), initiating transcription of *luxCDABEG* by activation of the *PBen* promotor (3), leading to quantifiable luminescence (4).

The *cocE* gene has previously served as the core entity in a cell-free protein expression system described by Voyvodic et al. [16], along with the benzoic acid receptor gene (*benR*) and the *PBen* promotor, using the native ribosome binding site (RBS) from *Pseudomonas putida*. The same components were employed in the sensing circuit reported herein, harboring the following activation cascade in an *Escherichia coli* host (Figure 1): CocE cleaves cocaine to ecgonine methyl ester and benzoate; the latter forms a complex with BenR, which binds to *PBen*, thereby activating the *Photobacterium leiognathi luxCDABEG* bioluminescence gene cassette [17]. The intensity of the elicited bioluminescent response is thus dependent on cocaine concentration. This design lays the foundation for the construction and testing of a cocaine-specific sensor; future generations of this construct will be characterized by an extended capacity for the detection of cocaine derivatives.

## 2. Materials and Methods

### 2.1. Chemicals

Cocaine HCl, fentanyl citrate, and ketamine (Klorketam), kindly provided by Prof. Ami Citri of the Hebrew University of Jerusalem, were dissolved in water and kept at room temperature. Cannabinoids (THC, CBD), kindly provided by Prof. Joseph Tam of the Hebrew University Medical School, were dissolved in ethanol and stored at −20 °C. Norketamine and Na benzoate were purchased from Sigma-Aldrich Israel Ltd (Rehovot, Israel), dissolved in methanol and stored at −20 °C (norketamine) or in water and kept at 4 °C (benzoate). Restriction enzymes *Sal*I, *Not*I, and *Xma*I and *Phusion* High-Fidelity DNA Polymerase were purchased from New England Biolabs (Ipswich, MA, USA) and stored at −20 °C. A 2× Taq master mix from Lambda Biotech (Ballwin, MO, USA) was used for colony PCR and stored at −20 °C.

### 2.2. Bacterial Strain

*E. coli* K12 strain DH5α (*NEB* #C2987H) was used as the host for all of this study’s plasmids and for the whole-cell sensor systems. DH5α cells were grown overnight at 37 °C with 200 rpm shaking in lysogeny broth (LB) containing 5 g/L yeast extract, 10 g/L tryptone, and 5 g/L sodium chloride, supplemented with ampicillin (100 μg/mL) and/or chloramphenicol (30 μg/mL). For long-term preservation, overnight cultures were mixed with glycerol (final concentration 25%) and stored at −80 °C.

### 2.3. Plasmid Design and Construction

PCR primers employed for the amplification of DNA segments from plasmids included functional sites for restriction enzyme recognition or DNA ligation using Gibson assembly, following the manufacturer’s protocol (NEBuilder HiFi DNA Assembly Cloning kit, New England Biolabs, Ipswich, MA, USA) [18]. Newly constructed plasmids were inserted into competent DH5α cells by a 1 min 42 °C heat shock. Plasmid DNA was purified using NucleoSpin^®^ Plasmid EasyPure (Macherey-Nagel, Dueren, Germany). Primers employed and/or constructed in the course of this study are listed in Table S1. The various molecular components employed in plasmid construction are listed in Table 1.

Cocaine sensor plasmids

The cocaine sensing circuit spanned two separate plasmids (Figure 1A,B):

I. Plasmid *pCocE-benR* contained the genes encoding the cocaine esterase enzyme (*cocE*) and the *P. putida* transcription factor gene *benR*. The *benR* sequence was amplified from plasmid *pBEAST-BenR* (a gift from Dr. Jerome Bonnet, CNRS, France; Addgene plasmid #114597) and was inserted into plasmid *pSB4C5_J23101-CocE* (a gift from Dr. Jean-Loup Faulon, INRAE, France; Addgene plasmid #128129).
II. The second plasmid, *pBen::luxPleio*, harbored a fusion of the *P. leiognathi luxCDABEG* gene cassette to the BenR-inducible *P. putida* promotor *PBen*. It is based on the C55_luxPleio plasmid described by Shemer et al. (2020), the *yqjF* gene promotor in which was replaced by double restriction enzyme digest with the *PBen* gene promoter using Gibson assembly.

Benzoate sensor plasmid

III. A third plasmid (pBen::luxPleio, Figure 1C,D) was constructed for the purpose of improving Pben responses to benzoate by accelerated evolution. The plasmid included the benR gene sequence, along with the *E. coli* arabinose-inducible expression system, composed of the PBAD promoter and its regulatory gene araC [19]. The benR sequence was amplified from plasmid pCocE-benR (this work), and those of araC and PBAD were amplified from plasmid PBAD-mTagBFP2 (Addgene cat. 34632). The vector backbone was digested with SalI, and the PCR products were assembled in the digested vector pBen::luxPleio using Gibson assembly.

**Table 1:** DNA components employed in the construction of cocaine and benzoate detection circuits.

Component	Origin	Description
*cocE*	*Rhococcus* sp.	*E. coli*-optimized cocaine esterase; cleaves cocaine into ecgonine methyl ester and benzoate.
*benR*	*P. putida*	Regulatory gene of the *ben* operon, activated by benzoate [20].
*PBen*	*P. putida*	The *ben* operon promoter, induced by a BenR/benzoate complex.
*luxCDABEG*	*P. leiognathi*	Bioluminescence gene cassette of *Photobacterium leiognath*i; *luxA* and *luxB* encode the heterodimeric luciferase, *luxCDE* a fatty acid reductase complex, and *luxG* a flavin reductase [21].
*araC*	*E. coli*	Regulatory gene of the arabinose operon [19].
*P_BAD_*	*E. coli*	Promotor of the arabinose operon, activated by arabinose [19].
*PBen*2	*P. putida*	Mutated *PBen* (this work).

### 2.4. Bacterial Sensor Strains

The sensor strains constructed in the course of this study are listed in Table 2. Cocaine sensor (CocS) cells were generated by co-transformation of plasmids *pCocE-benR* (chloramphenicol resistance) and *pBen::luxPleio* (ampicillin resistance) into competent DH5α cells. CocE (cocaine esterase) breaks down cocaine into ecgonine methyl ester and benzoic acid. BenR forms a complex with benzoic acid, which activates the *PBen* promotor [16]. This activation initiates the expression of the *lux* genes, subsequently leading to dose-dependent luminescence (Figure 1). The benzoate sensor (BenS) was constructed by transformation of plasmid *pBR-araBAD:benR-pBen::luxPleio* (ampicillin resistance) into DH5α cells. External addition of 6.6 mM arabinose drives *benR* expression via the *P_BAD_–araC* system. Benzoate complexes with BenR, leading to the expression of the *lux* cassette (Figure 1). Both CocS and BenS sensor strains were stored at −80 °C in 25% glycerol. CocS2 and BenS2 harbor a modified version of the *PBen* promoter (*PBen2*), generated by error-prone PCR as described in Section 2.7 below.

### 2.5. Luminescence Assay

Sensor strains were removed from −80 °C storage and grown overnight in 2 mL LB, supplemented with the appropriate antibiotics at 37 °C with shaking (200 rpm). The overnight CocS culture was diluted 100-fold into fresh LB and regrown under the same conditions for ca. 75 min to early exponential growth phase (OD_600_ ≈ 0.2). The BenS overnight culture was diluted 150-fold with LB containing 6.6 mM arabinose and regrown for 2 h. Aqueous aliquots (100 μL per well) of the target compound (cocaine HCl or Na benzoate, 0 to 2 mg/L) were added to individual wells of an opaque white 96-well plate with a transparent bottom (Greiner Bio-One, Kremsmünster, Austria). For the specificity test of the cocaine sensor strain, 5 mg/L cocaine, 40 mg/L fentanyl, 40 mg/L ketamine, 5 mg/L benzoate, 0.1 mg/L tetrahydrocannabinol (THC) and 2 mg/L cannabidiol (CBD) were used in aqueous solutions (100 μL per well). An aliquot (100 μL) of refreshed bacterial culture was added to each well, the plate was covered with a transparent cover, and luminescence was measured every 10 min for 12 to 16 h at 30 °C in a Tecan Infinite^®^ 200 PRO (Männedorf, Switzerland) plate reader. Luminescence values are depicted in the plate reader’s arbitrary relative luminescence units (RLUs) or as the ratio of the maximum luminescence intensity of the induced sample to that of the uninduced control (response ratio).

### 2.6. Cocaine Detection by CocS Paper Strips

Paper strips imbued with lyophilized CocS cells were designed for testing a simplified “dipstick”-type concept for rapid cocaine testing. CocS cells were grown overnight in 25 mL LB containing 100 μg/mL ampicillin and 30 μg/mL chloramphenicol to an optical density at 600 nm of ca. 2.5. Cells were pelleted by a 6 min centrifugation (3220 x g, 20 °C, Eppendorf 5810R) and resuspended in a prewarmed (37 °C) sterile protectant medium adapted from Stocker et al. [22] containing (*w*/*v*) 0.5% peptone, 0.25% yeast extract, 10% gelatin, 1% sodium ascorbate, 5% raffinose, and 5% glutamate. A 5 μL drop of cell suspension was placed on a marked spot of a 0.5 cm × 2 cm filter paper strip (Whatman #3). The strips were incubated for 5 min at room temperature and then transferred to a lyophilizer (Epson 1–4 LSCplus-CHRIST) and freeze-dried to −20 °C using the following program: 5 min at 1000 mbar, 2 h at 4 mbar, 2 h at 0.4 mbar, and 2 h at 0.04 mbar. Following the 6 h freeze-drying program, the lyophilizer was reset to 1000 mbar, and the paper strips were removed and stored at 4 °C.

For cocaine testing, paper strips were removed from refrigeration and, following equilibration to room temperature, were dipped into a cocaine solution (0 to 2 mg/L) for 30 min at 30 °C. The strips were removed from the samples, and luminescence was imaged in the dark for up to 5 h at 30 °C using a Sony Alpha 7sII camera with an exposure time ranging from 5 to 15 s. To prevent drying of the cells during storage, paper strips were either wrapped in a plastic foil or placed in a sealed plastic test tube.

### 2.7. Random Mutagenesis of the PBen Gene Promoter

To generate a library of *PBen* promoter mutants, the *PBen* region within the *pBR-araBAD:benR-pBen::luxPleio* plasmid was subjected to error-prone PCR using primers listed in Table S1. Taq polymerase was induced to introduce sequence errors into the amplification products by supplementing the PCR mixture with Mn^2+^ ions (250–375 μM as MnCl_2_), known to interfere with polymerase activity and to reduce its fidelity [23]. Fragment ends were functionalized by PCR primers for re-legation with the original plasmid. Plasmid *pBR-araBAD:benR-pBen::luxPleio* was pre-digested with *Not*I and *Xma*I restriction enzymes to remove the *PBen* promotor, generating a promotor-less, linearized plasmid. Purified mutated *PBen* PCR fragments were re-ligated into the promotor-less plasmid using Gibson assembly and transformed into *E. coli* strain DH5α to generate a library of approximately 1000 BenS variants. The library was screened for improved clones by inducing the cells with 0.5 mg/L benzoate and comparing the ensuing luminescence intensity with that of the benzoate-free control. Opaque white 384-well microtiter plates with a transparent bottom were prepared to contain 25 μL of either 0 mg/L or 0.5 mg/L benzoate in water per well, and 20 μL of a pre-grown and arabinose-activated BenS variant was added to each well. Luminescence and optical density at 600 nm were determined every 30 min for 15 h at room temperature. Promotor variants exhibiting an enhanced signal intensity and an increased response ratio compared with the parental plasmid were selected for further testing by exposure to a broader range of benzoate concentrations.

### 2.8. Calculations

The response ratio was determined by dividing the maximal luminescence (RLU) of the specific compound concentrations within the experimental course of 16 h with the maximal luminescence (RLU) of the compound-free control.

(1) Response ratio=RLU¯max, i ±SEMiRLU¯max, CTRL±SEMCTRL

where *i* is the respective concentration of the compound of interest, and *CTRL* describes the noise. Values are presented in the mean and the standard deviation of the mean (SEM). Error propagation according to Gauss was included in these calculations. Response data were analyzed by using the non-linear, four-parameter fit function of Origin (Academic) software (Version 2022b) with the Levenberg-Marquardt iteration logarithm. The chi-square tolerance value was set to 10^−9^.

The limit of detection (LOD) of the generated sensor strains was calculated as the average signal intensity of the noise (the luminescence signal of the uninduced controls) plus three standard deviations of the noise.

(2) $$LOD={\bar{x}}_{CTRL}+3\times {SEM}_{CTRL\;}\;$$

where ${\bar{x}}_{CTRL}$ is the mean of the maximal luminescence of the noise, and ${SEM}_{CTRL\;}$ is the standard deviation of the noise.

Data from independent experiments were combined by calculating the weighted average. The best estimate $x_{wav}$ was set as

(3) $$x_{wav}=\frac{\sum w_{i}x_{i}}{\sum w_{i}}$$

and the uncertainty $\sigma_{wav}$ of the weighted average

(4) $$\sigma_{wav}=\frac{1}{\sqrt{\sum w_{i}}}$$

with the weighting $w_{i}$ factor as

(5) $$w_{i}=\frac{1}{\sigma_{i}^{2}}$$

## 3. Results

### 3.1. Cocaine Detection by CocS

**Figure 2:**
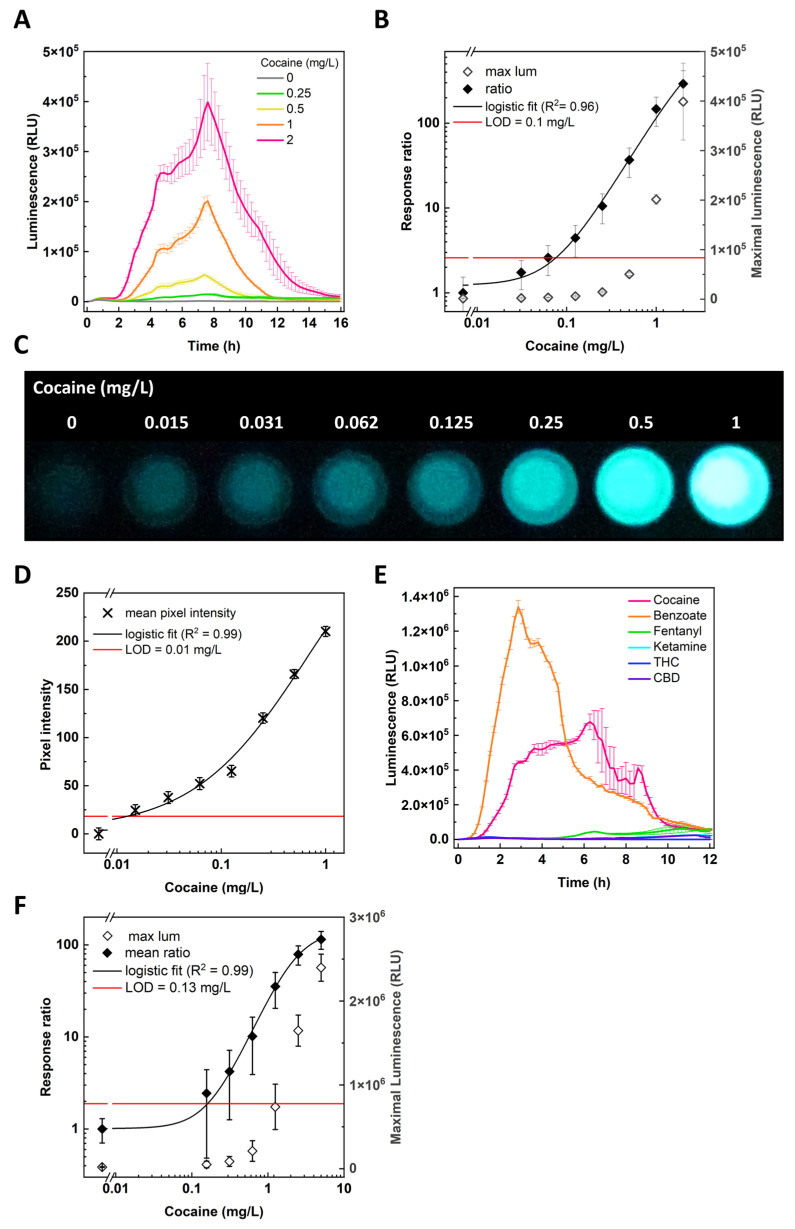
Luminescent cocaine detection by CocS cells in liquid medium. (**A**) Time course of luminescence development following exposure of CocS cells to the indicated cocaine concentrations. (**B**) Dose dependency of the luminescent signal (maximal values over a 16 h exposure), displayed both as signal intensity (◊) and as the response ratio (♦). Luminescence intensity values (mean ± SEM, *n* = 6) in both panels are presented in the plate reader’s arbitrary relative luminescence units (RLUs). LOD was determined using the response ratio plus three times the standard deviation of the background. (**C**) Luminescence of CocS cells 5.5 h post exposure to different cocaine concentrations. Image was captured by a Sony Alpha 7sII camera (4 s exposure, f-number 2, ISO 40,000). (**D**) Increase in pixel intensity of images shown in C compared with the cocaine-free control. Intensities were determined using the mean gray value measurement in ImageJ. Data shown represent mean ± SD (area = 3268 pixels) with incorporation of error propagation. LOD was set to be three standard deviations above the background. (**E**) Response specificity: luminescence development over time during exposure of CocS cells in the presence of different compounds (mean ± SEM, *n* = 2)—5 mg/L cocaine, 5 mg/L benzoate, 40 mg/L fentanyl, 40 mg/L ketamine, 0.1 mg/L THC, and 2 mg/L CBD. (**F**) Functionality in human urine: maximal luminescence presented both as signal intensity (◊) and as the response ratio (♦) of two individual samples from different donors (mean ± SEM, *n* = 2) with cocaine concentrations ranging from 0.152 to 5 mg/L.

CocS sensor strains, harboring plasmids *pCocE-BenR* and *pBen::luxPleio*, were exposed to a concentration series of cocaine as described under Materials and Methods, and luminescence was recorded every 10 min for 16 h. For concentrations above 0.25 mg/L, luminescence peaked after 8 h, with an apparent small shoulder after 4 h (Figure 2A). Figure 2B displays the maximal signal obtained in the course of the experiment, depicted both as the response ratio and as luminescence intensity, as a function of cocaine concentration. From photographic images of individual wells from a 96-well microtiter plate where lower concentrations were used (Figure 2C) and the pixel intensity of these images (Figure 2D), it may be observed that already at a concentration of 0.015 mg/L the luminescent signal appeared to be visually discernable from the cocaine-free control, displaying an apparent increase in detection sensitivity compared with the plate reader data. The calculated cocaine limits of detection (LODs) for both assays were indeed different—0.1 ± 0.1 mg/L from the plate reader data (Figure 2B) and 0.01 ± 0.02 mg/L from the image-derived data (Figure 2D). This 10-fold difference may be explained by the long exposure (4 sec) of the photographic image compared with the much shorter integration time (0.1 sec) of the plate reader. As shown in panel E, CocS cells luminesced only in the presence of cocaine and benzoate; no luminescence was observed in the presence of fentanyl (40 mg/L), ketamine (40 mg/L), THC (0.1 mg/L), and CBD (2 mg/L), highlighting the cocaine specificity of the assay. The tested concentrations of these compounds were selected following preliminary experiments, which determined them to be the highest non-toxic concentrations for the *E. coli* strain used. To test the sensor’s potential functionality in human body fluids, the same analytical protocol was employed on cocaine dissolved in urine. The data in Figure 2F exhibit dose dependency very similar to that observed in Figure 2B in an aqueous buffer, and a similarly low LOD (0.13 ± 0.05 mg/L). The reason for the higher error values at the lower cocaine concentrations is unclear; it may be due to differences in the urine composition in samples from different individuals.

### 3.2. Cocaine Detection by CocS Paper Strips

**Figure 3:**
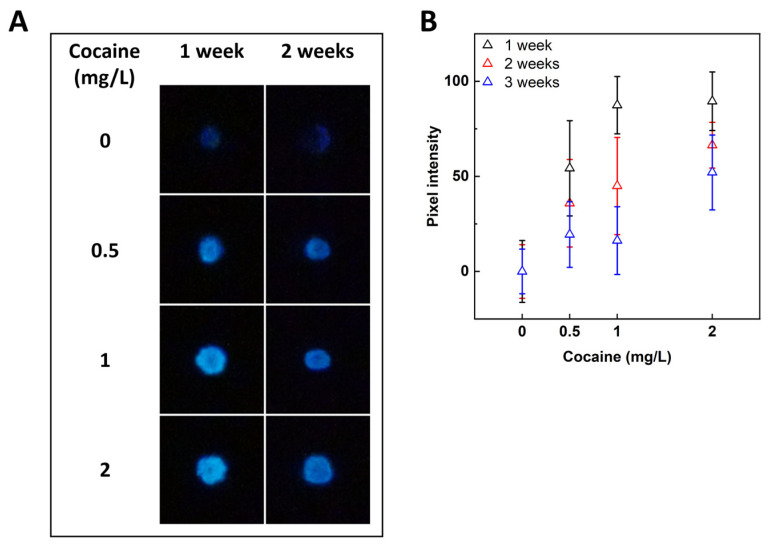
Induction of lyophilized CocS cells on paper strips by cocaine (30 min exposure, 30 °C). (**A**) Luminescence, imaged 5 h after induction using a Sony Alpha 7sII camera (15 s exposure, f-number = 2, ISO 40,000). (**B**) Increase in pixel intensity (compared with the cocaine-free control) of the images in panel A, analyzed using ImageJ software (version 1.54i, March 2024) which provided both the mean gray value and standard deviation within the bacterial spot region. The figure presents data from two independent experiments after storage at 4 °C. Values are presented in mean ± SEM.

CocS cells were lyophilized on paper strips to evaluate the potential applicability as a “dipstick”-type test. The bacteria-containing section of the strips was immersed in varying concentrations of cocaine (in H_2_O) for 30 min, air-dried, and imaged in the dark by a digital camera following a 5 h incubation at 30 °C. A clear luminescent signal was observed on the paper spots where CocS cells had been immobilized, indicating the presence of cocaine. Paper strips were stored at 4 °C and tested for cocaine detection functionality after 1, 2, and 3 weeks. Images for the first two weeks are shown in Figure 3A and the pixel intensities for the entire test period in Figure 3B. As demonstrated in Figure 3B, luminescence intensity decreased with incubation time but was nevertheless apparent even after 3 weeks of storage.

### 3.3. Benzoate Detection by BenS

The single-plasmid benzoate sensor strain constructed as described under Materials and Methods was employed as a chassis for screening the *PBen* variants emerging from its directed evolution process. The benzoate detection sensitivity of the parental BenS sensor was studied in both the presence and absence of arabinose. In the latter case, the expression of *benR* was not anticipated, and, consequently, no luminescence was expected.

**Figure 4:**
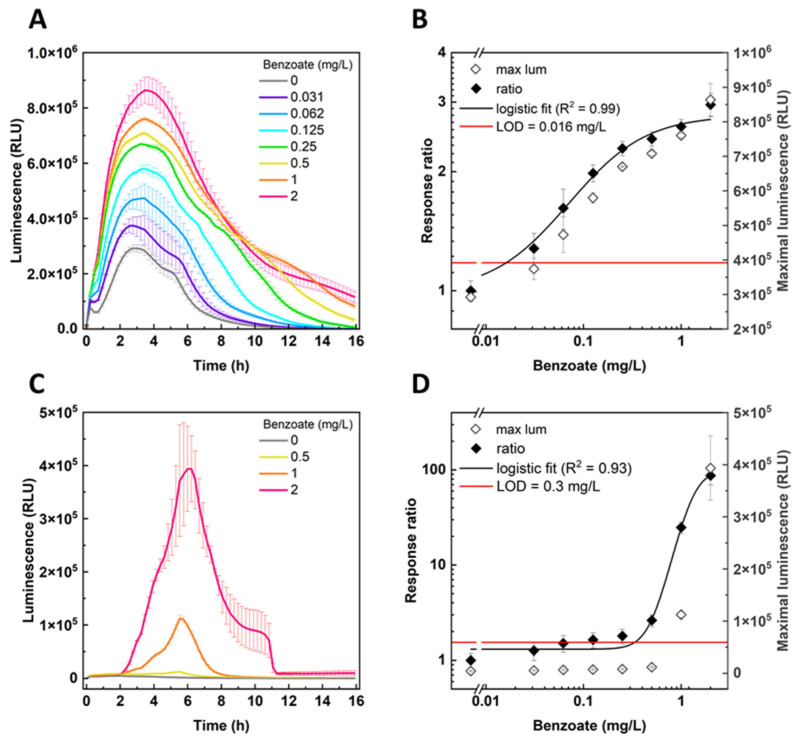
Induction of BenS sensor strain by benzoate. Bacteria were grown overnight at 37 °C and 200 rpm and diluted ×1/150 2 h prior to benzoate induction in the presence (**A**,**B**) or absence (**C**) of 6.6 mM arabinose. Luminescence (mean ± SEM, *n* = 3) was measured every 10 min at 30 °C. Luminescence values are in the plate reader’s arbitrary relative light units (RLUs). Panels (**B**,**D**) present the maximal luminescence (RLU) and calculated response ratio as a function of benzoate concentration in the presence or absence of arabinose, respectively.

As shown in Figure 4, the BenS strain responded in the presence of arabinose to all benzoate concentrations tested, from 31 μg/L to 2 mg/L, with luminescence peaking approximately 3 h post induction (Figure 4A). This response was earlier than that of the CocS sensor to cocaine (Figure 2A), as expected due to the lack of dependency on cocaine cleavage by cocaine esterase. The clear dose response (Figure 4B) allowed the calculation of the LOD, determined to be 0.016 ± 0.01 mg/L. Interestingly, there was a strong luminescent response to benzoate also without *benR* induction by arabinose (Figure 4C); this is likely due to a certain leakiness of the ***P****_BAD_* promoter, as has been previously shown for this element [19,24,25]. Dose response validation (Figure 4D) revealed a clear luminescence signal for benzoate concentrations higher than 0.5 mg/L but not for the lower concentration range. LOD was determined to be ca. 20 times higher (0.3 ± 0.12 mg/L) compared with the arabinose-induced experimental setup.

### 3.4. Directed Evolution of the PBen Promotor

**Figure 5:**
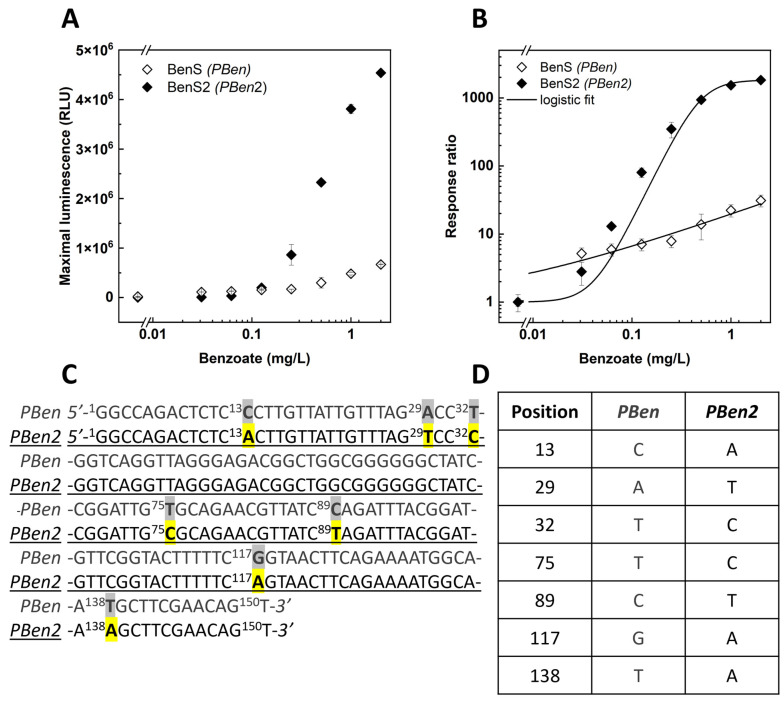
Responses to benzoate of the BenS2 sensor, harboring the mutated version of the *PBen* promoter (*PBen2*). (**A**) Maximal luminescence response (mean ± SEM, *n* = 3) of BenS2 and BenS to benzoate. (**B**) Response ratios of the two sensors. (**C**) Gene sequence of the *PBen* and *PBen2* promoters, with the seven point mutations highlighted. (**D**) Mutations in *PBen2* and their positions.

A single cycle of error-prone PCR amplification of the promotor region of the benzoate sensor yielded ca. 1,000 variants of the BenS sensor, which were then screened for their response to benzoate (0 and 0.5 mg/L). A small number of clones that displayed an improved performance compared with the parent construct, as evidenced by both the signal intensity and the response ratio, were analyzed for their response to a benzoate concentration series. The best performer out of these stood out by demonstrating a 7-fold increase in signal intensity (Figure 5A) and a remarkable 64-fold increase in the response ratio (in the presence of 2 mg/L benzoate; Figure 5B) when compared with the original BenS sensor. The new promoter variant was termed *PBen2*, and the sensor harboring it was termed BenS2. Results of the sequence analysis of this mutant revealed seven distinct point mutations at positions (Figure 5C,D).

### 3.5. Cocaine Detection with PBen2

**Figure 6:**
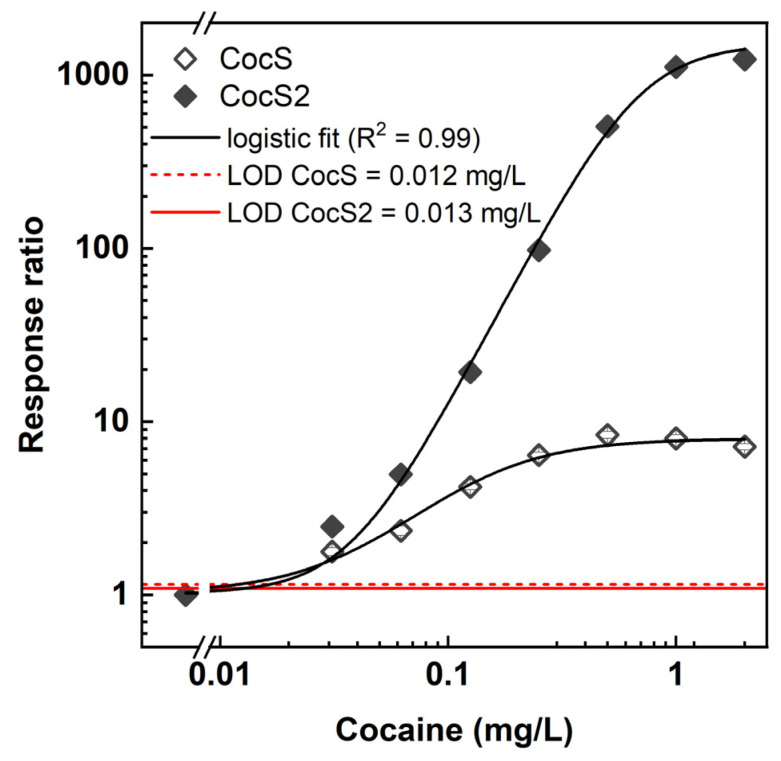
Cocaine detection (as response ratio) by CocS and CocS2 sensor strains.

The *PBen*2 promoter sequence was cloned into the *pBen::luxPleio* plasmid, which was then transformed into DH5α cells along with the second plasmid (*pCocE-benR)*, to generate a new cocaine sensor strain termed CocS2. Cocaine detection by this strain is summarized in Figure 6 along with those of the original CocS, demonstrating a notable enhancement in the response ratio. However, detection sensitivity remained similar, with calculated cocaine LODs of 12 ± 9 μg/L and 13 ± 9 μg/L for CocS and CocS2, respectively.

## 4. Discussion

The design and construction of microbial whole-cell biosensors (bioreporters) depends upon the identification of a genetic element (most often a gene promoter) inducible by the target compound and its fusion to reporter genes, the expression of which can be quantitatively monitored [1]. For the design of a cocaine bioreporter, we drew upon the report of Voyvodic et al. [16], who have used the production of benzoate by cocaine esterase (CocE) as a measure of cocaine concentration in a cell-free system. Based upon this report, we constructed a two-plasmid system that together generated a cascade involving a breakdown of cocaine to benzoic acid, the complexation of the latter with BenR, induction by this complex of the *PBen* gene promoter, and the luminescent expression of the *luxCDABEG* gene cassette. The sensor’s performance was further improved by modifying the sequence of the benzoate sensing element, significantly improving signal intensity but with no effect on detection sensitivity. The ability to distinguish cocaine from other substances was validated by testing it against a panel of commonly encountered drugs. Detection was demonstrated in liquid media as well as in urine, and its reproducibility was verified in several independent experiments. Table 3 summarizes the cocaine detection performance of the original (CocS) and mutated (CocS2) sensor strains. The CocS sensors displayed an averaged maximal response ratio of 7.3 ± 0.3, with luminescence values of (4.6 ± 1.8) × 10^4^ RLU in four independent experiments using cocaine dissolved in a buffer. The luminescent response peaked after ca. 8 h, but a discernable divergence from the background signal was apparent already after 2 h (Figure 2E). Measurable response in the paper strip assay was also obtained 1–2 h after exposure. Dose response evaluation revealed LOD values between 0.012 and 0.1 mg/L, with the camera achieving a LOD as low as 0.01 ± 0.02 mg/L cocaine using a long exposure time. The sensor was effective in human urine, though with a slightly higher LOD of 0.13 ± 0.05 mg/L cocaine. The mutated CocS2 strain exhibited a 30-fold increase in maximal luminescence signal intensity, a reduced background signal, and a 150-fold higher response ratio.

Furthermore, a proof of concept was presented for CocS application in a “dipstick”-type paper strip, which could be stored at 4 °C for 3 weeks without loss of functionality. The process of cocaine testing using CocS paper strips was as simple as using commercially available home-use point-of-care tests. For evaluation, a simple consumer-quality camera was required to assess the level of luminescence.

As mentioned in the Introduction, a major advantage of live cell-based sensors is their capacity to detect not only the original target molecule but also potential derivatives that share its biological activity. In the present communication, however, we concentrated on a cocaine-specific sensor and did not attempt to examine its ability to detect other molecules that share the same basic chemical structure. Such expended capacity will be the design objective of future generations of this bioreporter.

The cocaine concentration in the blood of addicts ranges between 0.5 and 1 mg/L, and concentrations around 4 mg/L were measured in cases of comatose-fatal doses [26,27]. The best limit of detection achieved with the optimized CocS2 (13 μg/L) not only is much lower than these values but can also compete with commercially available, point-of-care drug tests with cutoff levels between 10 and 20 μg/L cocaine [28]. Much lower concentrations of ca. 0.5 μg/L can be detected by laboratory-based ELISA or radioimmunoassays [29]. Similarly, detection limits of GC-MS and LC-MS techniques are in the range of 1 μg/L [30]. The cutoff level for cocaine detection in oral fluid, as defined by the U.S. Nuclear Regulatory Commission (NRC), is 15 μg/L [31]. This value is in the range of the CocS2 bioassay described herein, which does not require high-end laboratory equipment or specially trained personnel. The performance envelope presented in this study is thus in the detection range required by medical needs and is close to that characteristic of sophisticated bioanalytical methodologies. Additional molecular improvements in the construction of future generations of the CocS sensor are likely to push this envelope further.

In contrast to home-targeted drug testing kits, which are antibody-based immunological assays and thus invariably target specific chemicals, cell-based sensors offer a more versatile approach. A cell-based assay, which targets the biological effect of a drug rather than its chemical identity, is likely to detect additional chemicals with similar activities, such as derivatives of the same molecule. Such assays may thus be expected to provide at least a partial answer to the detection of new drug derivatives constantly appearing on the streets. Furthermore, as previously reported by Yagur-Kroll et al. [32], the response characteristics of a microbial sensor, such as signal intensity, response time, and detection threshold, can be significantly improved by directed evolution. We took a first step in this direction by the construction of the CocS2 sensor by a single round of error-prone PCR; while the process did not affect detection sensitivity, it nevertheless generated much stronger luminescence intensities, thus potentially reducing the need for high-performance optics. This directed evolution process will be repeated in the future for the construction of new and improved sensor strains, with an ability to also sensitively detect cocaine derivatives and analogs.

## Figures and Tables

**Table 2:** *E. coli* sensor strains constructed in the course of this study.

Sensor Strain	Plasmid	Functional Genes	Function
CocS	I. *pCocE-benR*	*cocE*, *benR*	Cleavage of cocaine into benzoate and activation of BenR
	II. *pBen::luxPleio*	*PBen*, *luxCDABEG*	Monitoring *PBen* activation by the benzoate–BenR complex
CocS2	I: *pCocE-benR*	*CocE*, *benR*	Cleavage of cocaine into benzoate and activation of BenR
	II: *pBen2::luxPleio*	*PBen2*, *luxCDABEG*	Monitoring *PBen*2 activation
BenS	*pBR-araBAD:benR-pBen::luxPleio*	*P_BAD_*, *araC*, *benR*, *PBen*, *luxCDABEG*	*benR* expression in the presence of arabinose and activation of BenR in the presence of benzoate. Monitoring *PBen* activation.
BenS2	*pBR-araBAD:benR-pBen2::luxPleio*	*P_BAD_*, *araC*, *benR*, *PBen2*, *luxCDABEG*	*benR* expression in the presence of arabinose and activation of BenR in the presence of benzoate. Monitoring of *PBen2* activation.

**Table 3:** Cocaine detection performance of sensor strains CocS and CocS2. Maximal luminescence and response ratio data are presented in mean ± SEM (*n* = 4) unless noted differently.

Sensor	Medium	Maximal Luminescence (RLU, 2 mg/mL Cocaine)	Maximal Response Ratio	LOD (mg/L)
CocS	Buffer	4.6 × 10^5^ ± 1.8 × 10^4^	7.3 ± 0.3	0.017 ± 0.008 ** (plate reader) 0.01 ± 0.02 (camera images)
CocS *	Urine	7 × 10^5^ ± 3 × 10^5^ (1.23 mg/L cocaine)	40 ± 15	0.13 ± 0.05
CocS2	Buffer	1.39 × 10^7^ ± 8 × 10^5^	1230 ± 80	0.13 ± 0.009

* Mean ± SEM (*n* = 2). ** A weighted averaged mean ± SEM from 4 independent experiments.

## Data Availability

All relevant data not included in the body of this article will be made freely available upon a request to the corresponding author.

## Supplementary Materials

The following supporting information can be downloaded at: https://www.mdpi.com/article/10.3390/s24206549/s1, Table S1: Plasmids and PCR primers constructed or employed in the course of this study [33,34,35].
