# Supplementary material for: A Microbial Cocaine Bioreporter
Source: Sensors (Basel). 2024 Oct 11;24(20):6549. doi: 10.3390/s24206549 (PMC11511522; doi:10.3390/s24206549)
Supplement: Supplementary file 1 [file sensors-24-06549-s001.zip › sensors-3212245-supplementary.pdf]

Table S1. Plasmids and primers used or constructed in the course of this study.

Plasmid	Primer	Function	Reference
pBEAST-BenR	*fw*: ACTATCGCACCATCAGCCAG<br>*rv:* GGCACCTGTCCTACGAGTTG	PCR amplification and restriction enzyme functionalization of *benR*	[1]
pSB4C5_J23101-CocE	*fw*: ATACGCAAGGCGACAA<br>*rv*: TATGCGTTCCGCTGTT	PCR amplification and restriction enzyme functionalization of *cocE*	[2]
pBR322-2TTS-C55::luxPleio	-	Plasmid harboring the *luxCDABEG* gene cassette	[3]
pBR322-2TTS-pBen::luxPleio	*fw:* TTGGGGATCGGGATCCCCGGGACTGTTCGAAGC<br>*rv:* ACTTTGGGTGATGATAGGGATA	Second plasmid of CocS	This work
pCocE-benR	*fw*: TTCTCCATACACAGAATCACCAAAAAGGAATAG<br>*rv:*<br>CTAACCAGTAAGTAATTACTTGAGATTCTCACCAATAAAA ACGCC	PCR amplification of *benR* for insertion into *pBen::luxPleio*	This work
P_{BAD}-mTagBFP2	*fw*:<br>TCGTTTTATTTGATGCCTGTCGACTTATGACAACTTGACGGCTAC<br>*rv:*<br>GTGATTCTGTGTATGGAGAAACAGTAGAGAGTTG	PCR amplification of ***P*_{BAD}**-*araC* gene, insertion of ***P*_{BAD}**-*araC* in *pBen::luxPleio*	[4]
pBR-pBAD:benR-pBen::luxPleio	*fw:*<br>TTTGCCCCTCCTGCGG<br>*rv:*<br>GATCGGGATCCCCGGG	Error-prone PCR of *PBen*	This work
pBR-pBAD:benR-pBen2::luxPleio	*fw*:<br>TTTGCCCCTCCTGCGG<br>*rv:*<br>GATCGGGATCCCCGGG	PCR amplification of *PBen2* for insertion into *pBen::luxPleio*	This work
pBR322-2TTS-pBen2::luxPleio	-	One of two plasmids of CocS2	This work

References

[1] Voyvodic, P.L.; Pandi, A.; Koch, M.; Conejero, I.; Valjent, E.; Courtet, P.; Renard, E.; Faulon, J.-L.; Bonnet J. Plug-and-play metabolic transducers expand the chemical detection space of cell-free biosensors. *Nat. Commun.* **2019**, *10*, 1697.

[2] Pandi, A.; Koch, M.; Voyvodic, P.L.; Soudier, P.; Bonnet, J.; Kushwaha, M.; Faulon, J.-L. Metabolic perceptrons for neural computing in biological systems. *Nat. Commun.* **2019**, *10*, 3880.

[3] Shemer, B.; Shpigel, E.; Glozman, A.; Yagur-Kroll, S.; Kabessa, Y.; Agranat, A.J.; Belkin, S. Genome-wide gene-deletion screening identifies mutations that significantly enhance explosives vapor detection by a microbial sensor. *N. Biotechnol.* **2020**, *59*, 65–73.
[4] Subach, O.M.; Cranfill, P.J.; Davidson, M.W.; Verkhusha, V.V. An enhanced monomeric blue fluorescent protein with the high chemical stability of the chromophore. *PLoS One* **2011**, *6*, e28674.
